# Juvenile Hyaline Fibromatosis: Literature Review and a Case Treated With Surgical Excision and Corticosteroid

**DOI:** 10.7759/cureus.10823

**Published:** 2020-10-06

**Authors:** Omar Braizat, Saif Badran, Atalla Hammouda

**Affiliations:** 1 Department of Plastic Surgery, Hamad Medical Corporation, Doha, QAT; 2 Department of Population Medicine, College of Medicine, QU Health, Qatar University, Doha, QAT

**Keywords:** hyaline fibromatosis, juvenile hyaline fibromatosis, plastic surgery, surgery, corticosteroid, corticosteroid treatment

## Abstract

Juvenile hyaline fibromatosis (JHF) is an extremely rare autosomal recessive disease with less than a hundred cases reported worldwide and is more prevalent in the middle east due to higher rates of interfamilial marriages. Manifestations can be debilitating, and patients typically present with decreased joint mobility, gingival hypertrophy, nodular skin lesions, papulonodular skin lesions and osteolytic bone disease. JHF is a relatively mild presentation of the hyaline fibromatosis syndrome (HFS) family of diseases, with Infantile hyaline fibromatosis (IHF) being the more lethal form. A mutation of the (CMG2) gene on chromosome 4q21 is hypothesized to result in the abnormal deposition of amorphous hyaline substance in different body tissues. There are few studies that evaluated the role of surgery, corticosteroid therapy and physiotherapy or a combination of these modalities in providing symptomatic relief. In our paper, we present a literature review and case presentation for 28-year-old women with JHF, treated with surgical excision and corticosteroid therapy. Early surgical treatment provided instantaneous and more sustainable results, while corticosteroids can be used as alternative modalities with temporary outcomes.

## Introduction

Hyaline fibromatosis syndrome (HFS) is a connective tissue disease with two different clinical manifestations of similar pathophysiology: infantile hyaline fibromatosis (IHF) and juvenile hyaline fibromatosis (JHF) [1-4]. The hallmark signs and symptoms of both variants are decreased joint mobility (95.2%), gingival hypertrophy (92.9%), skin lesions (85.7%) and osteolytic bone lesions (42.1%-68.4%) [1,3,5].

JHF was first described by Murray in 1873 and named molloscum fibrosum [6]. It is an extremely rare disease with around 84 cases reported world-wide and an autosomal recessive pattern of inheritance [5]. Incidence is equal among both genders [7]. JHF is associated with papulonodular skin lesions, large subcutaneous swellings commonly in the scalp and osteolytic lesions of the long bones, skull and phalanges [3,4,8]. This variant is usually discovered during early childhood and patients tend to have a relatively longer life span up to the fourth decade in most cases [9].

IHF is the more severe form with appearance of symptoms in the newborn period. It is characterized several signs and symptoms such as skin lesions, subcutaneous mass, gingival hyperplasia, as well as more systemic involvement such as short stature, joints stiffness and diffuse osteopenia [3,4]. Higher mortality rate is noticed and is caused by failure to thrive, severe diarrhea and septicemia [4,5,10].

A few articles briefly described promising outcomes of surgical intervention [2,3,11,12] and questionable results after corticosteroid therapy [2,3,13] for skin manifestations in patients with HFS. We present a case of JHF treated for numerous large skin nodules with a combination of surgical excision, corticosteroid therapy and physiotherapy.

## Case presentation

A 28-year-old Sudanese woman with a bachelor’s degree in English literature was diagnosed with JHF during her childhood, her parents are cousins of the second degree. She has two sisters, one of whom shares the same diagnosis. The patient’s initial presentation was in the form of a facial papular skin rash. She was 1.5 years of age and living with her parents in her home country of Sudan at the time. At the age of five years, she developed skin nodules and gingival hypertrophy, a skin biopsy was taken and showed a periodic acid-Schiff (PAS) positive myxoid background with scattered spindle cells, confirming the diagnosis of JHF. By her late teenage years, disease progression continued, and bilateral knee and elbow stiffness started emerging.

Upon attending the genetics clinic in Hamad General Hospital (HMC), Doha, Qatar at 20 years of age, she had joint stiffness, multiple masses on her hands and typical skin features. She underwent multiple excision biopsy procedures for the various subcutaneous swelling in her back and was following with the endocrine and gastrointestinal clinics for short stature, vitamin-D deficiency, chronic diarrhea and malabsorption. She was 34 kg in weight and 150 cm in height. DNA testing confirmed the diagnosis of JHF by detection of large homozygous deletions of exons 15 and 16 in the ANTXR2 gene.

In December 2016, the patient had her first encounter with the Plastic Surgery team. She presented with multiple small hard nodules over the forehead, nose, ears and big toe. Patient received local triamcinolone acetonide injections for numerous bilateral hand nodules at that time, and excision of other facial lesions. At one-year follow-up post-surgical removal, no recurrence of the facial lesions was noted. Patient also noted a significant decrease in pain, size, and softening of her hand skin lesions.

**Figure 1:**
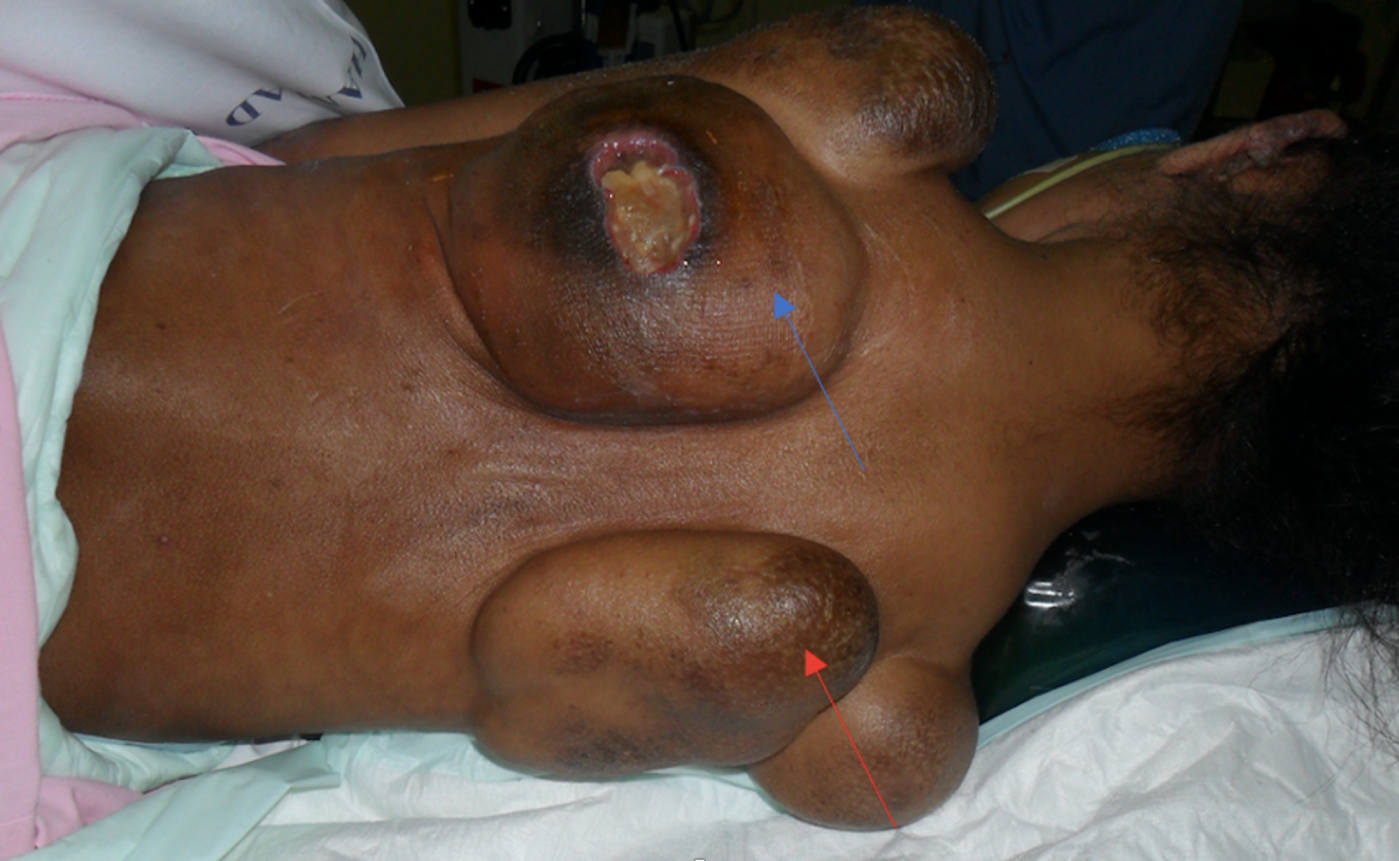
Preoperative photo showing two large back nodules. Left back lesions, soft, irregular edges, measuring around (8 x 8 x 7 cm), with skin ulceration (blue arrow). Right back lesion, soft, irregular edges, measuring around (8 x 8 x 7 cm), associated with hyperpigmented skin on top (red arrow).

In December 2017, two new lesions at the back, each measuring around (8 x 8 x 7 cm), respectively, were excised (Figure 1). Histopathology was again consistent with JHF. Post-operative course was smooth, and no recurrence was detected.

In February 2019, the left shoulder subcutaneous mass was increasing in size and the patient had a return of numerous, previously injected hand growths. The lesions were innumerable and local injections would cause significant pain. Hence, a trial of 40 mg oral prednisolone was attempted for one week and then half of the dose over two weeks, followed by a quarter of the original dose over another two weeks. Patient reported a noticeable improvement in pain, the size of lesions and joint stiffness while she was on oral corticosteroid therapy.

**Figure 2:**
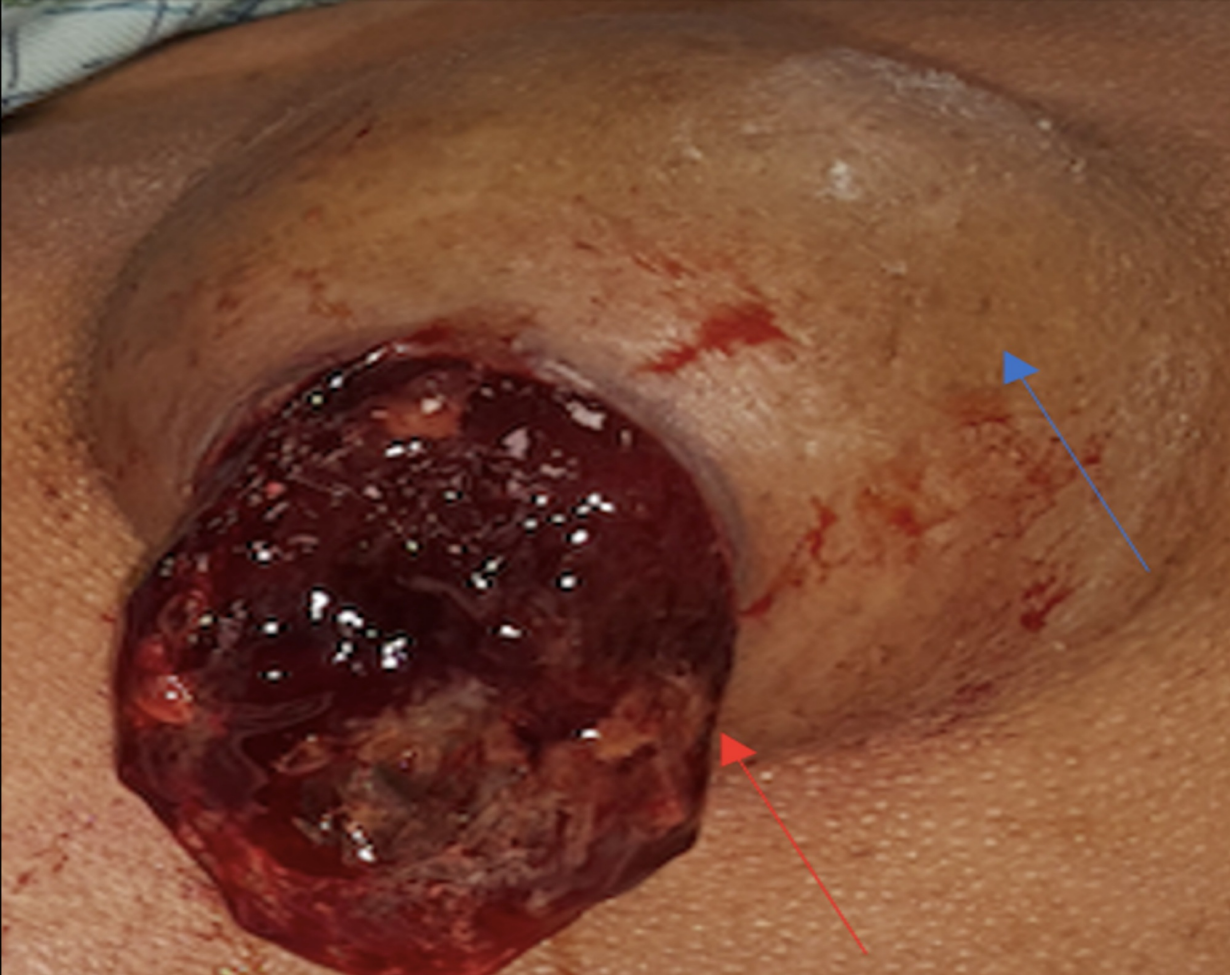
Gross appearance of the left shoulder nodule intraoperatively. Lesion measured around (6 x 8 x 7 cm), soft consistency, with ulceration (blue arrow). Thick mucinous material was extruded from the ulcerated site by itself without pressure or incision (red arrow). Skin incisions were made on both sides of the ulcer, and the nodule was excised along with excess skin to result in a linear wound after primary closure.

In May 2020, patient underwent excision of the enlarging left shoulder swelling (15 x 8 x 8 cm) with overlying skin necrosis and multiple bilateral ear and chin swellings. Grossly, lesions contained thick mucinous slough (Figure 2). She was satisfied with the outcome and no reappearance was detected at three months post operation.

**Figure 3:**
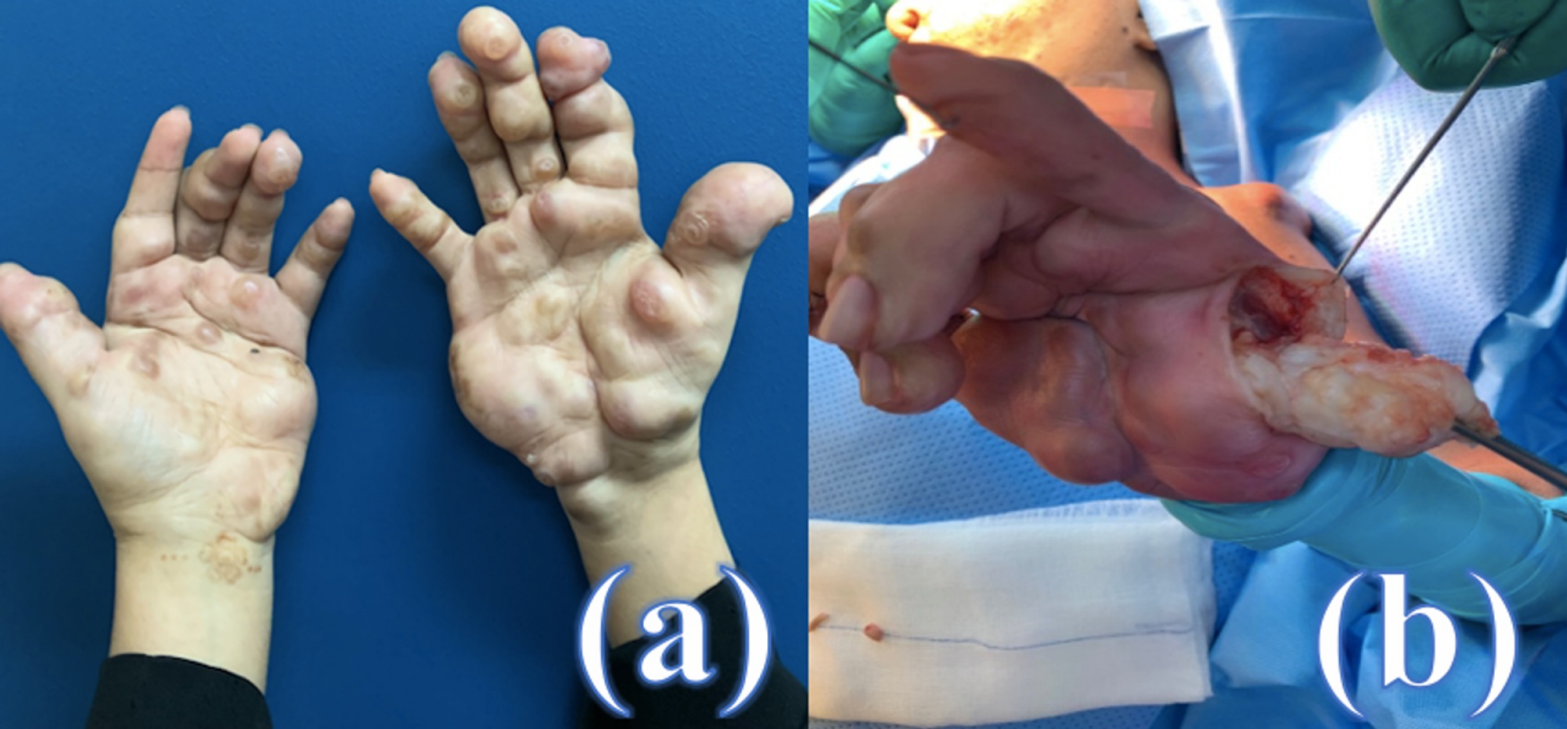
Preoperative photo showing multiple hard and painful hand nodules, with different sizes and locations on the palmar side (a). Intraoperative photo showing the fibrous and hard nature of the left-hand nodule, over the hypothenar (b). Numerous lesions were excised with a primary closure of the wounds.

In August 2020, patient follow-up showed no recurrence of the previously excised lesions in the shoulders and back. Hand nodules that were treated by local and systemic corticosteroid showed only partial and temporary improvement. She was operated to remove some of the remaining painful hand lesions to improve her function and relieve the pain (Figure 3). 

## Discussion

Currently, literature is considering the presence of a spectrum of presentations rather than two distinct diseases: IHF and JHF. This is supported by the fact that both diseases share identical histopathologic findings and similar genetic mutations [1-3]. Dowling et al suggested that missense, truncating, and frameshift mutations, affecting the extracellular von Willebrand A (vWA) domain, are associated with infantile systemic hyalinosis (ISH), whereas in-frame and missense mutations are associated with the phenotypically milder JHF [10]. HFS has a high prevalence among middle east and north African populations, areas where intrafamilial marriage and parent consanguinity is a common finding [5,14].

Most genetic studies identified a mutation in the capillary morphogenesis factor-2 gene (CMG2), also known as ANTXR2 gene (Anthrax Toxin Receptor 2) located on chromosome 4q21. This gene codes for a transmembrane cell receptor that participates in strengthening and supporting connective tissue by reacting with the extracellular matrix [5,15,16]. This mutation translates into the allelic disorders JHF and ISH [1,2,10].

Under light microscopy, there is a deposition of hyaline-like material in many tissues such as skin, skeletal muscle, gastrointestinal tract lymph nodes and other tissues with the exception of brain tissue, which explains the common finding of normal mental development [1,3-5,15]. Typical features on electron microscopy include a dilated rough endoplasmic reticulum and hypertrophic Golgi apparatus filled with fibrillar and granular material, commonly displacing the nucleus and rest of the cytoplasm [3,5,8,13]. The epidermis is normal while the papillary and reticular dermis shows deposits of eosinophilic and PAS-positive homogenous material in the extracellular and perivascular spaces [1-5,13].

There is no consensus in the literature regarding the pathophysiology of HFS. It has been suggested that increased synthesis of glycosaminoglycans by fibroblasts might be the cause. Another possible culprit is the increase in type VI and III collagen, along with increased degradation of type I collagen, which can explain the clinical features of inflexible skin and the limitation of joint movement [3,5,7,8,16].

Our patient had skin lesions in paired musculoskeletal sites like shoulders and hands, which is a common finding in this condition [17-19]. JHF usually affects certain anatomical locations like nose, ears and extremities [11,12,15,20]. The consistency of the subcutaneous nodules can vary from soft to hard, and sometimes associated with skin ulceration [7].

Regarding treatment modalities for this debilitating disease, no clear guidelines are available and supportive care is the mainstay of management at the present time. We have attempted a combined approach using intralesional corticosteroid injections, systemic corticosteroid and surgical excision of large skin nodules followed by physiotherapy. 

Our patient had multiple excisional surgeries with no recurrence of the same growths and a dramatic and instantaneous improvement in the cosmetic and functional status. Whether surgery is performed for cosmetic [8] or functional purposes, early excision is recommended from childhood [2,11,12]. However, the reoccurrence of lesions has been reported [3,5,17]. 

As previously reported by El-Maytaah et al, local triamcinolone injection resulted in symptomatic relief [20]. From our experience, local injections are a valid treatment option in small nodules within the proximity of vital structures that are not amenable to surgical excision. After Injection, the patient described temporary improvement in size, pain and consistency, which resulted in improved hand function. However, local injections are not feasible when skin manifestations are extensive due to the painful nature of the procedure. Such cases may benefit from oral corticosteroid therapy that might be more beneficial if given for more than seven days like our case. 

Although physiotherapy can play a role in treating the joints stiffness associated with JHF; however, not all patients will have a significant improvement. Our patient reported no noticeable change. We believe it should be used in combination with other treatment modalities and not as solitary treatment.

## Conclusions

JHF is a progressive connective tissue disease associated with multiple skin nodules that can cause functional and cosmetic impairment. Early surgical treatment is of a higher benefit and results in sustainable outcomes. The use of local and systemic corticosteroid can be used as alternative modalities of treatment with temporary results.
